# High fat diet-induced TGF-β/Gbb signaling provokes insulin resistance through the *tribbles* expression

**DOI:** 10.1038/srep30265

**Published:** 2016-08-03

**Authors:** Seung-Hyun Hong, Moonyoung Kang, Kyu-Sun Lee, Kweon Yu

**Affiliations:** 1 Neurophysiology and Metabolism Research Group, Korea Research Institute of Bioscience and Biotechnology (KRIBB), Daejeon 34141, Korea; 2 Functional Genomics Dept., University of Science and Technology (UST), Daejeon 34113, Korea; 3 Convergence Research Centre for Dementia, Korea Institute of Science and Technology (KIST), Seoul 02792, Korea

## Abstract

Hyperglycemia, hyperlipidemia, and insulin resistance are hallmarks of obesity-induced type 2 diabetes, which is often caused by a high-fat diet (HFD). However, the molecular mechanisms underlying HFD-induced insulin resistance have not been elucidated in detail. In this study, we established a *Drosophila* model to investigate the molecular mechanisms of HFD-induced diabetes. HFD model flies recapitulate mammalian diabetic phenotypes including elevated triglyceride and circulating glucose levels, as well as insulin resistance. Expression of *glass bottom boat* (*gbb*), a *Drosophila* homolog of mammalian *transforming growth factor*-β (*TGF-β*), is elevated under HFD conditions. Furthermore, overexpression of *gbb* in the fat body produced obese and insulin-resistant phenotypes similar to those of HFD-fed flies, whereas inhibition of Gbb signaling significantly ameliorated HFD-induced metabolic phenotypes. We also discovered that *tribbles*, a negative regulator of AKT, is a target gene of *Gbb* signaling in the fat body. Overexpression of *tribbles* in flies in the fat body phenocopied the metabolic defects associated with HFD conditions or Gbb overexpression, whereas *tribbles* knockdown rescued these metabolic phenotypes. These results indicate that HFD-induced TGF-β/Gbb signaling provokes insulin resistance by increasing *tribbles* expression.

Obesity, defined as an excessive accumulation of lipid in fat tissue, is associated with an elevated risk of developing insulin resistance and metabolic abnormalities, including diabetes and cardiovascular disease^1^. Adipose tissue is not only the primary site for storage of excess nutrients, but also functions as an endocrine organ by secreting numerous cytokines, growth factors, and hormones that regulate metabolic homeostasis^2^,^3^. Obese individuals have high circulating levels of adipokines, adipose tissue–derived cytokines that contribute to the development of metabolic dysfunctions and inflammatory responses^4^,^5^,^6^. The adipokine transforming growth factor-β (TGF-β) was recently identified as a critical mediator of insulin resistance in obesity-induced metabolic diseases. Circulating TGF-β levels are significantly elevated in obese humans, *ob/ob* mice, and high-fat diet (HFD)-induced obese mice^7^,^8^. By regulating expression of its target genes, such as PGC-1α and PPAR-γ, elevated TGF-β/Smad3 signaling is associated with systemic insulin resistance and hepatic steatosis^8^,^9^. Systemic neutralization or inhibition of TGF-β in HFD-induced obese mice ameliorates these phenotypes, suggesting that TGF-β signaling makes a physiologically relevant contribution to the progression of metabolic disease^10^. However, the molecular mechanism underlying the link between TGF-β signaling in adipose tissue and the development of insulin resistance has not been elucidated.

The *Drosophila* genome contains a compact set of TGF-β signaling factors including seven ligands, four type I receptors, two type II receptors, and four Smad proteins. Therefore, *Drosophila* is regarded as a versatile model system for the study of TGF-β signaling^11^. The *Drosophila* protein Glass Bottom Boat (Gbb) is a TGF β family member that regulates growth, differentiation, and tissue morphogenesis^12^,^13^,^14^. *gbb* mutant larvae are transparent due to the reduction of lipid contents in the fat body, the functional counterpart of mammalian adipose and liver tissue^15^. Gbb signaling affects several aspects of metabolism and energy homeostasis. For example, fat-derived Gbb remotely controls the expression of neuronal *Drosophila insulin–like peptide 2* (*Dilp2*) and regulates the transcription of genes involved in amino acid uptake, lipid hydrolysis, and the transport of these molecules in the fat body^16^. Because many metabolic pathways, including insulin signaling^17^,^18^,^19^, lipid metabolism^20^,^21^, and food intake^22^,^23^, are conserved between *Drosophila* and mammals, we used the *Drosophila* model to investigate the role of TGF-β/Gbb signaling in conditions of nutrient excess or obesity.

HFD-fed flies exhibited abnormal glucose and lipid levels and insulin resistance similar to those observed in obese mammals. The HFD-induced insulin resistance was mediated by the activity of the Gbb–tribbles pathway in the fat body. Thus, targeted inhibition of Gbb–tribbles signaling represents a new therapeutic strategy for treatment of obesity and its associated metabolic diseases.

## Results

### Induction of *gbb* expression mimics HFD phenotypes in *Drosophila*

Overgrowth of adipose tissue increases release of TGF-β family ligands in mammals^8^,^24^, but it remains unclear how these ligands are involved in metabolic phenotypes. To investigate the functions of TGF-β family ligands in metabolic disease, we fed adult *w*^*1118*^ flies a HFD containing 20% coconut oil for 14 days. As in mammals, dietary fat induces obesity and diabetic phenotypes in *Drosophila*. Whole-body triglyceride (TG) levels started to increase after HFD feeding: the TG level peaked 4 days after initiation of HFD and was maintained until day 14 (Figure S1A, top). The trehalose/glucose level did not change during the first 4 days, but started to increase on day 6 and reached a maximum at day 10 (Figure S1A, bottom). Expression of *Dilp2* mRNA and secretion of the encoded protein were also increased by HFD feeding, peaking on day 4 and decreasing to control levels between day 6 and day 10 (Figure S1B). In flies subjected to long-term HFD, insulin-stimulated AKT phosphorylation (pAKT) in the fat body was significantly lower than in control flies (Figure S1C).

**Figure 1:**
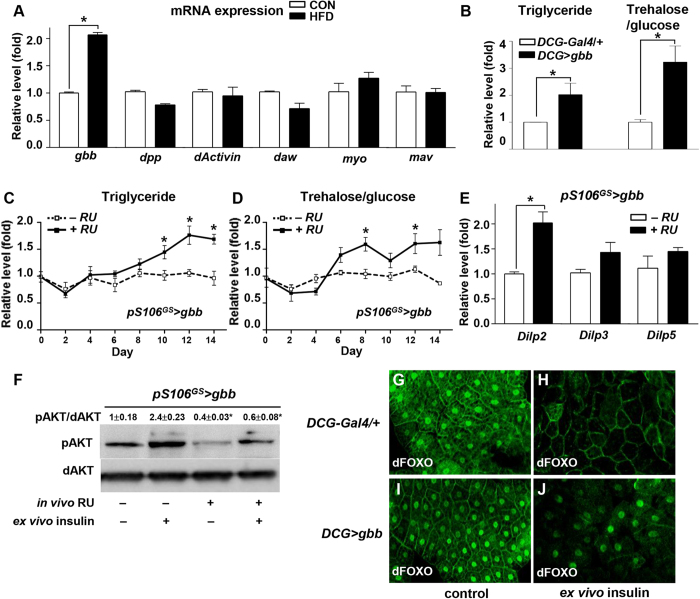
Induction of *gbb* in the fat body regulates metabolic phenotypes and insulin signaling. (**A**) Expression levels of TGF-β ligands in HFD. *gbb* expression was increased in flies fed HFD relative to the level in flies fed a normal control diet. (**B**) Levels of triglyceride and trehalose/glucose in DCG > *gbb* were elevated relative to those in the *DCG-Gal4* control. (**C, D**) During 14 days of HFD feeding, levels of triglyceride and trehalose/glucose in *pS106*^*GS*^ > *gbb* + RU gradually increased relative to those in the −RU control. (**E**) *pS106*^*GS*^ > *gbb* + RU increased *Dilp2* expression relative to the level in the −RU control. (**F**) *pS106*^*GS*^ > *gbb* + RU with or without insulin suppressed pAKT activation relative to that in the −RU control. (**G–J**) In the larval fat body, dFOXO was mainly localized in the nuclei in the *DCG-Gal4* control (**G**) and in *DCG* > *gbb* (**I**). After insulin treatment of *ex vivo* cultured larval fat bodies, dFOXO was localized in the cytoplasm of the *DCG-Gal4* control (**H**), but most dFOXO remained in nuclei in *DCG* > *gbb* (**J**). Data are presented as means ± s.e.m. from at least three independent experiments. *P < 0.05.

On day 14 of the HFD, we measured the expression levels of seven ligands of the TGF-β superfamily in the adult fly fat body. Of the factors we tested, only *gbb* expression was significantly upregulated by HFD feeding (Fig. 1A), particularly in the fat body (Figure S2A). Next, we investigated whether *gbb* was able to change the levels of TG in the fat body and trehalose/glucose in the hemolymph. *gbb* overexpression in the adult fat body (*DCG* > *gbb)* increased the levels of TG and trehalose/glucose compared with those in control flies (*DCG-Gal4/ + *) (Fig. 1B). However, *gbb* overexpression in the gut (*NP3084* > *gbb*) or muscle (*MHC* > *gbb*) did not change the level of TG (Figure S2B). Furthermore, the level of pAKT was significantly reduced in the fat-derived *gbb* (*DCG* > *gbb*) but not in the gut- or muscle-derived *gbb* (Figure S2C).

To determine whether induction of *gbb* in the adult flies mimics the effects of a HFD, we used the inducible gene switch *pS106*^*GS*^*-Gal4* driver, which can be activated by the addition of the mifepristone/RU-486 (RU)^25^. *pS106*^*GS*^*-Gal4* driver induced *gbb* expression only in the adult fat body (Figure S2D). Over 14 days, the levels of TG and trehalose/glucose gradually increased in flies overexpressing *gbb* in the adult fat body (*pS106*^*GS*^ > *gbb*, + RU) relative to those in control flies (*pS106*^*GS*^ > *gbb*, −RU) (Fig. 1C,D). These results demonstrate that HFD-induced obesity can activate *gbb* in the adult fat body, resulting in hyperlipidemia and hyperglycemia.

*gbb* overexpression in the adult fat body (*pS106*^*GS*^ > *gbb* + RU) significantly increased expression of *Dilp2*, and slightly increased expression of *Dilp3* and *Dilp5*, relative to the levels in −RU controls (Fig. 1E). To determine whether *gbb* regulates insulin signaling, we dissected adult fat body from *pS106*^*GS*^ > *gbb* + RU or −RU flies and treated them with insulin *ex vivo* (Figure S2E). In the −RU controls*, ex vivo* insulin treatment increased the level of pAKT relative to that in untreated samples. By contrast, in samples obtained from + RU flies, which overexpressed *gbb* in the adult fat body, *ex vivo* insulin treatment did not increase the level of pAKT (Fig. 1F).

To further investigate the regulation of insulin signaling by *gbb*, we examined the subcellular localization of dFOXO in larval fat bodies using the *DCG-Gal4* fat body–specific driver. In these experiments, dissected fat bodies were serum-starved and treated with human insulin *ex vivo*. In the starved *DCG-Gal4* control and *DCG* > *gbb* fat bodies, dFOXO was mainly localized in the nuclei (Fig. 1G,I). Upon insulin stimulation, almost all dFOXO protein was re-localized to the cytoplasm in *DCG-Gal4* control fat bodies (Fig. 1H), whereas in *DCG* > *gbb* fat bodies most dFOXO protein was still located in nuclei (Fig. 1J). These results indicate that *gbb* overexpression in the fat body inhibits insulin signaling, resulting in an insulin-resistant phenotype similar to that observed under long-term HFD feeding.

### Inhibition of Gbb signaling rescues HFD-induced obesity and diabetic phenotypes

**Figure 2:**
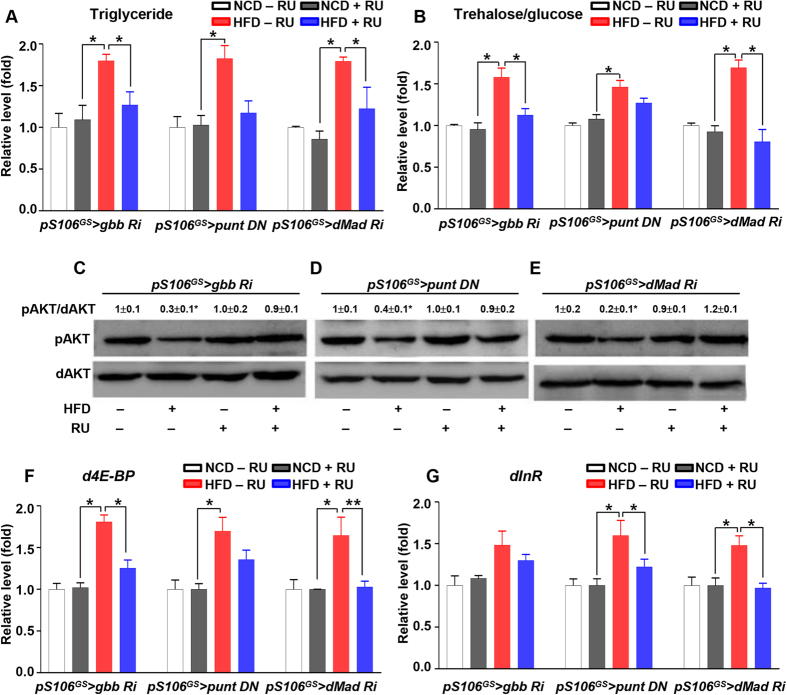
Inhibition of Gbb signaling rescues HFD-induced obesity and diabetic phenotype. (**A,B**) In the HFD condition, *pS106*^*GS*^ > *gbb RNAi* + RU, *pS106*^*GS*^ > *punt DN* + RU, *pS106*^*GS*^ > *dMad RNAi* + RU suppressed the elevated levels of triglyceride and trehalose/glucose observed in −RU to control levels (i.e., the levels in flies fed a normal control diet). (**C–E**) In the HFD condition, *pS106*^*GS*^ > *gbb RNAi* + RU, *pS106*^*GS*^ > *punt DN* + RU, *pS106*^*GS*^ > *dMad RNAi* + RU restored the reduced pAKT levels observed in −RU to control levels. (**F,G**) In the HFD condition, *pS106*^*GS*^ > *gbb RNAi* + RU, *pS106*^*GS*^ > *punt DN* + RU *pS106*^*GS*^ > *dMad RNAi* + RU suppressed the elevated expression of *d4E-BP* and *dInR* observed in −RU to control levels. Data are presented as means ± s.e.m. from at least three independent experiments. *P < 0.05.

Next, we tested whether inhibition of Gbb signaling would rescue HFD-induced obesity and the insulin-resistant phenotype. Gbb signaling in the fat body was inhibited by *gbb RNAi* (*DCG* > *gbb Ri*), dominant negative (DN) form of type I receptors (*DCG* > *sax DN*, *DCG* > *tkv DN*), type II receptor (*DCG* > *punt DN*), or Gbb signaling transducer *dMad RNAi* (*DCG* > *dMad Ri*). The elevated TG level in the HFD *DCG-Gal4/* + control was not observed in *DCG* > *gbb Ri*, *DCG* > *sax DN*, *DCG* > *punt DN*, and *DCG* > *dMad Ri* (Figure S3A). To confirm this result only in the adult fat body, we used RU-inducible gene switch system *pS106*^*GS*^*-Gal4* and inhibited Gbb signaling by *pS106*^*GS*^ > *gbb RNAi*, *pS106*^*GS*^ > *punt DN*, or *pS106*^*GS*^ > *dMad RNAi*. In the normal control diet (NCD) condition, inhibition of Gbb signaling did not change the levels of TG or trehalose/glucose relative to those in −RU controls. However, in the HFD condition, inhibition of Gbb signaling suppressed the elevation of TG and trehalose/glucose levels observed in the absence of RU (Fig. 2A,B). Next, we examined insulin signaling in the fat body by monitoring AKT activation. HFD feeding decreased the pAKT level in −RU controls, but this reduction in pAKT was restored when Gbb signaling was inhibited (HFD, + RU) (Fig. 2C–E). Elevated expression levels of dFOXO target genes *d4E-BP* and *dInR* in the HFD condition were also restored when Gbb signaling was inhibited (HFD, + RU) (Fig. 2F,G). Thus, inhibition of Gbb signaling in the fat body rescued HFD-induced obesity and insulin resistance. To further test the insulin-resistance phenotype under the HFD condition, we dissected fat bodies from flies in which Gbb signaling was inhibited and treated them with insulin *ex vivo*. The insulin-resistance phenotype (reflected by pAKT levels) in the HFD, −RU, + insulin condition was rescued in the HFD, + RU (Gbb signaling inhibited), + insulin condition (Figure S3B,C,D). These data provide further evidence that inhibition of Gbb signaling in the fat body can rescue the insulin-resistance phenotype.

### *tribbles* is a downstream target of Gbb signaling

**Figure 3:**
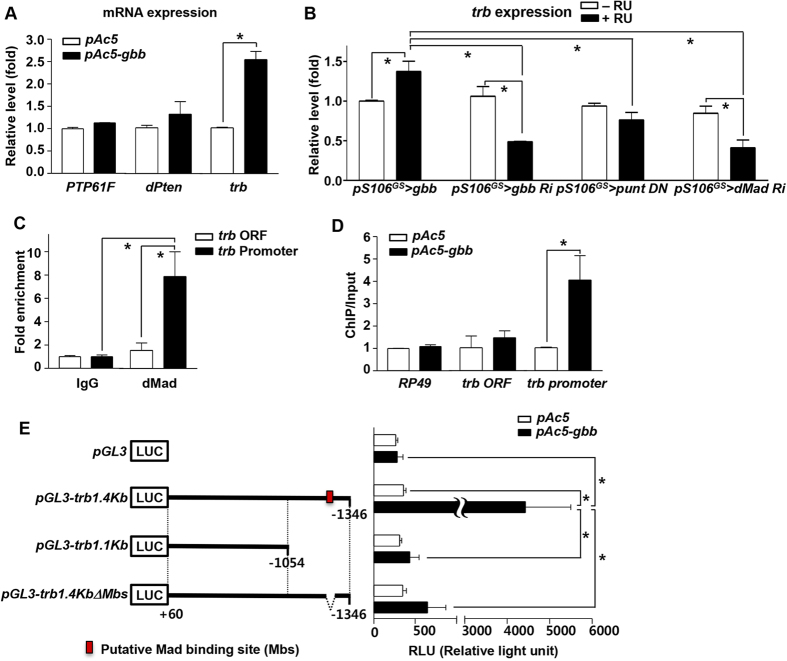
tribbles is a downstream target of Gbb signaling. (**A**) In *Drosophila* S2 cells, *gbb* transfection induced expression of *trb*, not *PTP61F* and *dPten*. (**B**) Expression levels of *trb* increased in the fat bodies of *pS106*^*GS*^ > *gbb* + RU flies relative to those in −RU controls, but decreased in *pS106*^*GS*^ > *gbb RNAi* + RU, *pS106*^*GS*^ > *punt DN* + RU, and *pS106*^*GS*^ > *dMad RNAi* + RU. (**C**) In *Drosophila* S2 cells, dMad was associated with the *trb* promoter region, which contains putative dMad-binding sites, but not with the *trb* coding region (ORF). (**D**) *gbb* transfection enriched dMad binding at the *trb* promoter region, but had no effect on binding by nonspecific IgG (negative control). (**E**) Schematic representations of the *pGL3* luciferase reporter vector (LUC) and *trb* upstream genomic DNA fragments containing the putative Mad binding site (red box) (left) and the luciferase activities by *gbb* overexpression (right). The significantly increased luciferases activity with *pGL3-trb1.4kb* was not observed with *pGL3-trb1.1kb* and *pGL3-trb1.4kbΔMbs*. Data are presented as means ± s.e.m. from at least three independent experiments. *P < 0.05.

To determine how Gbb signaling negatively regulates insulin signaling, we measured the expression levels of negative regulators of insulin signaling in *gbb*-transfected cultured *Drosophila* S2 cells. *Protein Tyrosine Phosphatase 61F* (*PTP61F*) and *dPten* decrease the activity of dInR and phosphoinositide 3-kinase (PI3K), respectively^19^,^26^, and Tribbles-related protein (Trb) inhibits pAKT^27^,^28^. In *gbb*-overexpressing S2 cells, the expression level of *trb*, but not *PTP61F* and *dPten*, was higher than that in controls (Fig. 3A). *trb* expression was also elevated in fat body from *gbb*-overexpressing adult flies (*pS106*^*GS*^ > *gbb* + RU), but reduced in the fat body of flies in which Gbb signaling was inhibited (*pS106*^*GS*^ > *gbb RNAi*, *pS106*^*GS*^ > *punt DN*, *pS106*^*GS*^ > *dMad RNAi* + RU), relative to the expression level in −RU controls (Fig. 3B). Together, these *in vitro* and *in vivo* results demonstrate that *trb* is a downstream target gene of Gbb signaling.

To determine whether *trb* is a direct transcriptional target of Gbb signaling, we performed chromatin immunoprecipitation (ChIP) assays with dMad antibody, followed by PCR amplification of *trb* regulatory sequences, in *Drosophila* S2 cells. In particular, we amplified the putative promoter and the ORF of the *trb* gene, both of which contain potential Mad-binding consensus sequences (GTCT) (Figure S4A,B). In the ChIP-PCR assay, we observed an amplified band corresponding to the *trb* promoter, but no band corresponding to the ORF (Figure S4C). In a ChIP-qPCR assay, the *trb* promoter was enriched 8-fold with dMad antibody (Fig. 3C). In *gbb*-overexpressing S2 cells, the association level between dMad and the *trb* promoter was elevated, but this was not the case for the *trb* ORF (Fig. 3D). In the luciferase reporter assays, 1.4kb *trb* upstream genomic DNA fragment containing the putative Mad binding site increased the luciferase activity significantly when *gbb* was overexpressed. However, 1.1kb *trb* upstream genomic DNA fragment and 1.4kb *trb* upstream genomic DNA fragment missing only the putative Mad binding site didn’t increase the luciferase activity (Fig. 3E). These findings provide strong evidence that the Gbb signal transducer dMad directly binds to the *trb* promoter and regulates *trb* transcription.

### *tribbles* negatively regulates insulin signaling

**Figure 4:**
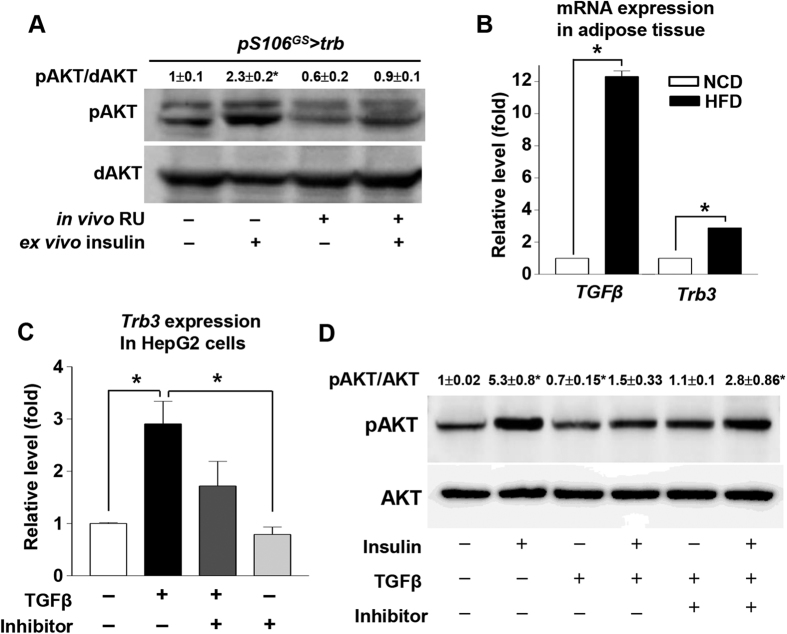
*tribbles* negatively regulates insulin signaling. (**A**) Overexpression of *trb* in the fat body (*pS106*^*GS*^ > *trb* + RU) decreased pAKT activation relative to that in −RU controls. (**B**) In mouse adipose tissue, HFD increased expression of *TGF-β* and *Trb3* relative to the corresponding levels in mice fed a normal control diet. (**C**) In HepG2 cells, TGF-β induced *Trb3* expression, which was restored by treatment with TGF-β receptor inhibitor. (**D**) TGF-β with or without insulin inhibited AKT activation, which was restored by treatment with TGF-β receptor inhibitor treatment. Data are presented as means ± s.e.m. from at least three independent experiments. *P < 0.05.

To determine whether Trb can inhibit pAKT in *Drosophila*, as it does in mammals, we measured pAKT levels in fat bodies from *pS106*^*GS*^ > *trb* flies. pAKT was increased by *ex vivo* insulin treatment in −RU control fat bodies, whereas, in *trb*-overexpressing fat bodies (*pS106*^*GS*^ > *trb*, + RU), the insulin stimulated pAKT level was not elevated relative to the no-insulin control (Fig. 4A). These results confirm that *tribbles* can inhibit pAKT in the *Drosophila* fat body. Likewise, in adipose tissue of HFD-fed mice, expression of *TGF-β* and *Trb3* was elevated relative to the corresponding levels in NCD-fed mice (Fig. 4B). To determine whether TGF-β signaling can induce *Trb3* expression and inhibit insulin signaling in mammals, we treated HepG2 cells with TGF-β ligand. *trb* expression was increased 3-fold over control levels by TGF-β treatment, but less than 2-fold by treatment with TGF-β and TGF-β receptor inhibitor (Fig. 4C). When we measured the pAKT level in HepG2 cells, insulin treatment alone increased the pAKT level by 5-fold relative to control, whereas treatment with insulin and TGF-β caused no increase in the pAKT level. Moreover, treatment with insulin, TGF-β, and TGF-β receptor inhibitor increased the pAKT level by 3-fold (Fig. 4D). These findings indicate that, in mammals, as in *Drosophila*, TGF-β signaling turns on the *Trb3* gene and negatively regulates insulin signaling by blocking pAKT.

### *tribbles* inhibition suppresses *gbb*-induced obesity and diabetic phenotype

**Figure 5:**
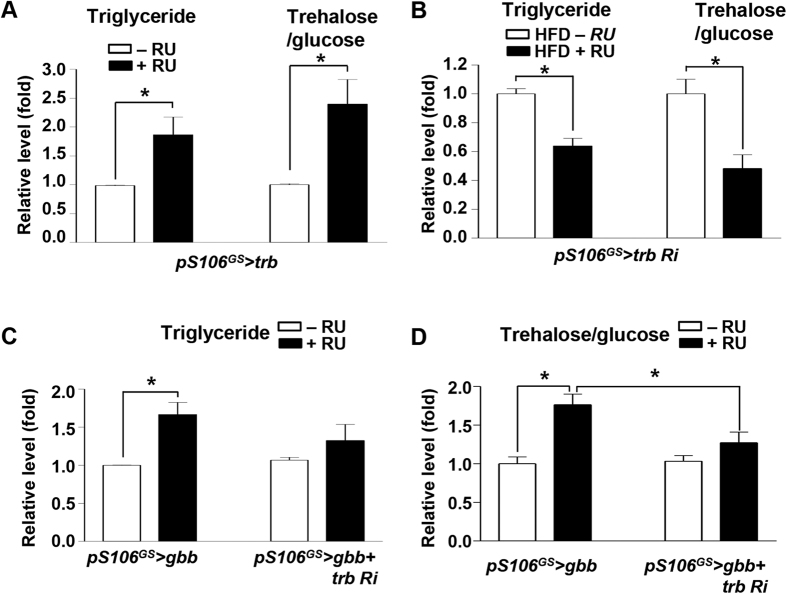
Inhibition of *tribbles* suppresses *gbb*-induced obesity and diabetic phenotype. (**A**) Levels of triglyceride and trehalose/glucose in *pS106*^*GS*^ > *trb* + RU increased relative to those in −RU controls. (**B**) In the HFD condition, knockdown of *trb* in the fat body suppressed the elevated levels of triglyceride and trehalose/glucose. (**C**,**D**) Knockdown of *trb* in *gbb*-overexpressing fat body (*pS106*^*GS*^ > *gbb* + *trb RNAi* + RU) suppressed the elevated levels of triglyceride and trehalose/glucose observed in *pS106*^*GS*^ > *gbb* + RU. Data are presented as means ± s.e.m. from at least three independent experiments. *P < 0.05.

As with *gbb*, expression of *trb* increased in HFD flies (Figure S5A; compare with Fig. 1A). Therefore, we investigated the metabolic roles of *trb*. When *trb* was overexpressed in adult fat body (*pS106*^*GS*^ > *trb* + RU), the levels of TG and trehalose/glucose were higher than those in −RU controls (Fig. 5A) and expression levels of dFOXO target genes *d4E-BP* and *dInR* were increased relative to those in −RU controls (Figure S5B). Inversely, when *trb* expression was knocked down in the adult fat body (*pS106*^*GS*^ > *trb RNAi* + RU), the levels of TG and trehalose/glucose were reduced (Fig. 5B). To determine whether Gbb signaling regulates metabolism through *trb*, we knocked down *trb* in the adult fat body of *gbb*-overexpressing flies (*pS106*^*GS*^ > *gbb + trb RNAi*) and monitored metabolic phenotypes. The levels of TG and trehalose/glucose in *pS106*^*GS*^ > *gbb + trb RNAi* + RU conditions were lower than those in *pS106*^*GS*^ > *gbb* + RU (Fig. 5C,D). Expression levels of dFOXO target genes *d4E-BP* and *dInR* were also lower in *pS106*^*GS*^ > *gbb + trb RNAi* + RU than in *pS106*^*GS*^ > *gbb* + RU (Figure S5C,D). These results strongly suggest that *gbb* regulates obesity and diabetic phenotypes by regulating *trb* expression in the fat body.

## Discussion

Abnormally high fat mass is a major risk factor for the development of diabetes. Previous studies emphasize that excess adiposity results in abnormal production of cytokines, growth factors, and hormones^4^,^5^, which in turn causes secondary diseases like insulin resistance^6^. In this study, we demonstrated that HFD-induced obesity triggered TGF-β signaling, which downregulates insulin signaling in the fat body. We also demonstrated the role of *tribbles*, a novel target of TGF-β/Gbb signaling, in the development of insulin resistance.

*Drosophila* models were used in several recent studies of diet-induced obesity, insulin resistance, hyperglycemia, and hyperinsulinemia^29^,^30^,^31^,^32^. In *Drosophila* larvae, a high-sugar diet induces type 2 diabetic phenotypes including hyperglycemia, high TG, and insulin resistance^31^. Likewise, in adult flies, HFD feeding also induces high TG and altered glucose metabolism, and in mammals it causes cardiac dysfunctions like diabetic cardiomyopathy^29^. In this study, we established a *Drosophila* model of obesity-induced insulin resistance, which has remarkable parallels with the mammalian system, and used it to observe and investigate the development of insulin resistance under chronic over-nutrition conditions. In addition, to study the *Drosophila* insulin-resistance phenotype in detail, we developed an *ex vivo* culture system (Figure S2E).

When we fed adult flies a HFD, their short- and long-term metabolic responses were different: for example, expression and secretion of Dilp2 was increased by short-term HFD but decreased by long-term HFD. Insulin signaling, which was assayed by monitoring pAKT activation and expression of the dFOXO target genes *d4E-BP* and *dInR*, was activated in short-term but not long-term HFD, whereas TG and trehalose/glucose levels in hemolymph were increased by long-term HFD (Figure S1). Because these pathological phenotypes in flies were very similar to the phenotypes associated with insulin-resistant diabetes in mammals, we conclude that HFD adult flies can be used as a model of type 2 diabetes.

In addition to increasing TG levels, HFD feeding in flies increased the expression of *gbb*. In mice, inhibition of TGF-β signaling by knockout of *Smad3* protects against diet-induced obesity and diabetes^8^. Inhibition of TGF-β signaling may improve adipose function and reverse the effects of obesity on insulin resistance. The TGF-β/Smad3 signaling also plays a key role in adipogenesis^33^. However, it remains unclear how TGF-β signaling is related to the onset of diet-induced obesity and diabetes. In this study, we examined the effects of *Drosophila* TGF-β family ligands on obesity. Of the genes we tested, only *gbb* was upregulated by HFD (Fig. 1A). Gbb regulates lipid metabolism and controls energy homeostasis by responding to nutrient levels^16^; consequently, *gbb* mutants have extremely low levels of fat in the fat body, resembling a nutrient-deprived phenotype^16^. On the contrary, *gbb* overexpression increased the TG level, mimicking the effects of nutrient-rich conditions (Fig. 1B). These data suggest that TGF-β/Gbb signaling is involved in HFD-induced obesity. Indeed, overexpression of *gbb* in the fat body phenocopied the TG and trehalose/glucose levels in flies fed a HFD (Fig. 1C,D compared with Figure S1A). However, *Dilp2* expression was increased by *gbb* overexpression in the fat body, consistent with the effects of short-term but not long-term HFD (Fig. 1E compared with Figure S1B).

We next focused on three negative regulators of insulin signaling, *PTP1b*, *PTEN*, and *tribbles* 3 (*TRB3*), which are involved in insulin resistance in obese mammals^34^,^35^,^36^. *tribbles* was upregulated in *gbb*-overexpressing cells and flies, (Fig. 3A,B). In mammals, *Tribbles* encodes an evolutionarily conserved kinase that plays multiple roles in development, tissue homeostasis, and metabolism^37^. A mammalian *Tribbles* homolog, *Tribbles homolog 3* (*TRB3*), is highly expressed in liver tissue under fasting and diabetic conditions, and inhibits insulin signaling by direct binding to Akt and blocking phosphorylation-dependent Akt activation^28^. Indeed, the expression level of *TRB3* is elevated in patients with type 2 diabetes and animal models of this disease^38^. In the systemic sclerosis model, TGF-β signaling can induce mammalian *TRB3* and activates TGF-β signaling-mediated fibrosis^39^. Recent work showed that *Drosophila tribbles*, like mammalian *TRB3*, inhibits insulin-mediated growth by blocking Akt activation^18^. In this study, *tribbles* expression was increased in HFD conditions in both mice and flies (Figs 4B, S5A), as well as in TGF-β–treated human liver cells (Fig. 4C). *tribbles* knockdown rescued the diabetic phenotypes caused by HFD (Fig. 5B), consistent with previous findings in mammals. In addition, *tribbles* knockdown rescued the diabetic phenotypes caused by *gbb* overexpression (Fig. 5C,D). These data strongly suggest that the evolutionarily conserved *tribbles* gene is a novel downstream target of Gbb signaling, and that *tribbles* knockdown rescues diabetic phenotypes in flies. Therefore, future studies should seek to elucidate TGF-β–Trb3 signaling and its functions in mammalian adipocytes; the resultant findings could suggest new strategies for preventing type 2 diabetes.

In summary, we established a *Drosophila* insulin-resistance model and demonstrated that Gbb signaling in the fat body plays a critical role in obesity-mediated insulin resistance by regulating *tribbles* expression. These results provide insights regarding the function of Gbb/TGF-β signaling in metabolic disease, and suggest that this pathway represents a promising therapeutic target for treatment of obesity and diabetes.

## Materials and Methods

### ***Drosophila melanogaster*** stocks

*Drosophila melanogaster* were cultured and kept at 25 °C on a NCD containing 38% cornmeal, 20% yeast, 5% sugar, and 2% agar. *w*^*1118*^ flies were obtained from the Bloomington Stock Center, *pS106*^*GS*^*–Gal4* from Dr. M. Tatar, *DCG-Gal4* from Dr. J. Graff, *UAS-gbb*, *UAS-dpp*, *UAS- UAS-dActβ*, and *UAS-punt DN* from Dr. M. O’Conner, and *UAS-tribbles* from Dr. M. Milán. We also obtained *UAS-gbb RNAi*, *UAS-dMad RNAi*, and *UAS-tribbles RNAi* lines from the Vienna *Drosophila* Resource Center. In order to express *pS106*^*GS*^*–Gal4*, adult flies were cultured on an agar-based diet supplemented with 200 μM RU486 (Sigma).

### Cell culture, stimulation, and transfection

*Drosophila* S2 cells purchased from Invitrogen were maintained at 26 °C in Schneider medium supplemented with 10% bovine calf serum. HepG2 cells were cultured in 4.5 g/l glucose Dulbecco’s modified Eagle’s medium (DMEM) supplemented with 10% fetal bovine serum, 2% L-glutamine, 100 mU/ml penicillin, and 100 mg/ml streptomycin in a 5% CO_2_ atmosphere at 37 °C. The culture medium was changed every 2–3 days. Before peptide treatments, cells were starved for 8 h in serum-free medium containing 0.5% BSA and pretreated with chemical inhibitor or vehicle. TGF-β and TGF-β RI kinase inhibitor II (10 mM, Calbiochem) were used in these experiments. For transfection, cells were cultured in growth medium without antibiotics and transfected with small interfering RNA (siRNA) using Xtreme GENE HP (Roche). For overexpression of *gbb*, the full-length *gbb* cDNA was cloned into vector pAc5 (Invitrogen).

### HFD and feeding

The HFD was prepared by the addition of 20% (vol/vol) coconut oil to the NCD; these proportions were used in all HFD experiments. The HFD was administered to male flies collected when they were 3–5 days old.

### Measurement of body TG level

Thirty adult fly bodies were collected and homogenized, and TG levels were measured using the Triglyceride Determination Kit (Sigma). TG levels were normalized to total protein levels.

### Measurement of trehalose/glucose level in hemolymph

Hemolymph was collected from 20 flies, and concentrations of trehalose/glucose were measured as previously described^40^.

### RNA purification and quantitative RT-PCR analysis

Fat bodies from 20 flies were collected for RNA preparation. Total RNA extraction and quantitative RT-PCR were performed as previously described^23^. mRNA levels were expressed as the fold change relative to the corresponding level of *rp49* mRNA, used as an internal normalization control. The comparative cycle threshold (Ct) method (User Bulletin 2, Applied Biosystems) was used to analyze the data. Primers used in this study are listed in Supplementary Table 1.

### *Ex vivo* culture of adult fat bodies

Adult fat bodies were dissected in the Schneider’s medium, starved for 8 h in serum-free medium containing 0.5% BSA, and incubated for 1 h in Schneider medium containing human insulin (100 nM, GIBCO).

### Western blot analysis and immunostaining

Total protein from adult fat bodies was isolated in *Drosophila* homogenate buffer (10 mM HEPES, 100 mM KCl, 1 mM EDTA, 5 mM DTT, 0.1% Triton X-100, 10% glycerol, and protease inhibitor cocktail [Sigma]). Western blots were performed as previously described^22^. Phospho-AKT and dAKT primary antibodies (both used at 1:1000) were obtained from Cell Signaling, and horseradish peroxidase–conjugated anti-rabbit IgG secondary antibody (1:2000) was obtained from Santa Cruz Biotechnology. Immunostaining was performed as previously described^41^ with dFOXO primary antibody (1:200; gift from Dr. M. Tatar) and the anti-rabbit IgG Alexa 488 secondary antibody (1:200, Molecular Probes).

### ChIP

Cells were fixed in 0.5% formaldehyde for 15 min. ChIP analysis was performed as described^23^. After nuclei were isolated and chromatin was sonicated, an aliquot of DNA was removed as the input control, and the remainder was incubated with either normal mouse IgG (2 μg, Santa Cruz Biotechnology) or mouse anti-dMad (2 μg, Santa Cruz Biotechnology). Traditional PCR and qPCR were performed using input DNA and immunoprecipitated DNA. Primers used in this study are listed in Supplementary Table 2.

### Luciferase reporter assay in *Drosophila* S2 cells

For luciferase assays, 1.4 kb *Drosophila trb* upstream genomic DNA (from +60 to −1346) and 1.1 kb (from +60 to −1054) were amplified by PCR, subcloned into the *pGL3* luciferase reporter vector (Promega), and generated *pGL3-trb1.4kb* and *pGL3-trb1.1kb*. *pGL3-trb1.4KbΔMbs*, the deletion construct of the putative Mad binding sequence (GACATCCGTCTG) in *pGL3-trb1.4Kb*, was generated by overlap extension PCR using primers sitting on flanking sequences of the deletion^42^. These DNA constructs and *pAc5.1A-gbb* or *pAc5.1A* protein expression vector were transfected in *Drosophila* S2 cells with Xtreme GENE HP (Roche). After 48 h, cells were harvested and measured the luciferase activity using Luciferase reporter assay system (Promega) according to manufacturer’s instructions. The assays were repeated three times. Primers used in this study are listed in Supplementary Table 2.

### Statistical analysis

Each experiment was repeated at least three times, and the data were presented as mean ± s.e.m. Student’s *t*-test was used for statistical analyses, and *P* < 0.05 was accepted as statistically significant.

## Additional Information

**How to cite this article**: Hong, S.-H. *et al*. High fat diet-induced TGF-β/Gbb signaling provokes insulin resistance through the *tribbles* expression. *Sci. Rep*. **6**, 30265; doi: 10.1038/srep30265 (2016).

## Supplementary Material

Supplementary Information
