# Supplementary material for: High fat diet-induced TGF-β/Gbb signaling provokes insulin resistance through the tribbles expression
Source: Sci Rep. 2016 Aug 3;6:30265. doi: 10.1038/srep30265 (PMC4971497; doi:10.1038/srep30265)
Supplement: Supplementary Information [file srep30265-s1.pdf]

# High fat diet-induced TGF-β/Gbb signaling provokes insulin resistance through the *tribbles* expression

Seung-Hyun Hong[1], Moonyoung Kang[1, 2], Kyu-Sun Lee[1, 2], and Kweon Yu[1, 2, 3]

[1] Neurophysiology and Metabolism Research Group, Korea Research Institute of Bioscience and Biotechnology (KRIBB), Daejeon 34141, Korea

[2] Functional Genomics Dept., University of Science and Technology (UST), Daejeon 34113, Korea

[3]Convergence Research Centre for Dementia, Korea Institute of Science and Technology (KIST), Seoul 02792, Korea

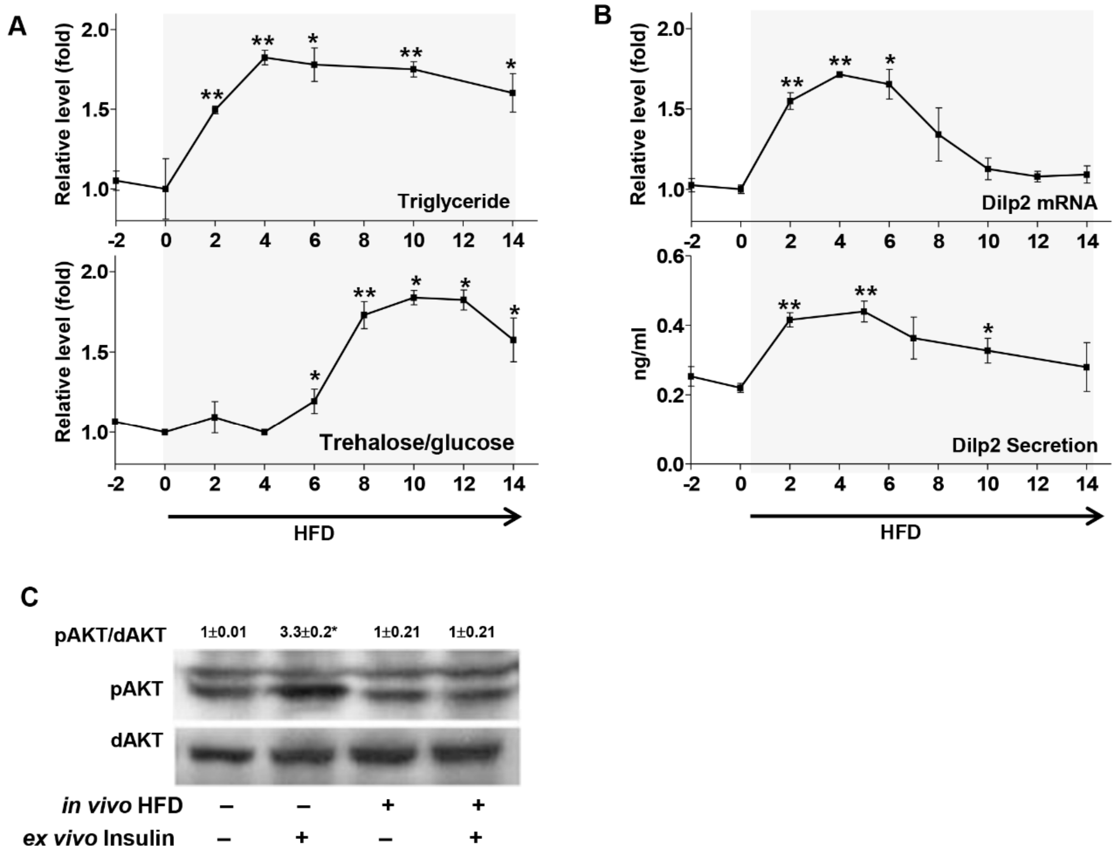


**Supplementary Figure 1. HFD induces obesity and diabetic phenotypes.** (A) Levels of triglyceride and trehalose/glucose after HFD feeding. (B) Levels of *Dilp2* mRNA and Dilp2 secretion after HFD feeding. (C) *Ex vivo* insulin treatment activated pAKT in the fat bodies of flies fed a normal diet, but not in those from flies fed a HFD. Data are presented as means ± s.e.m. from at least three independent experiments. *$P$<0.05.

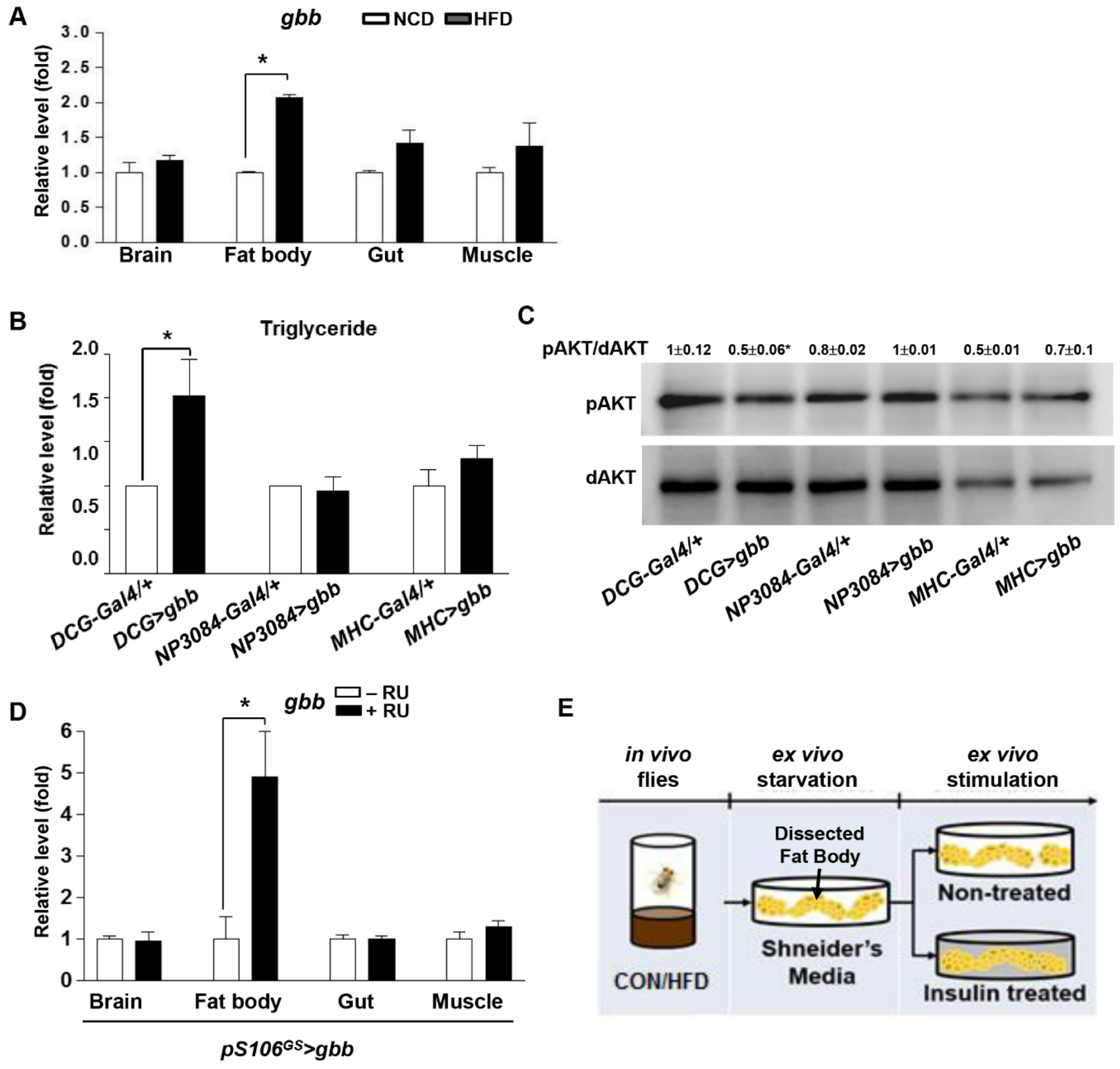


**Supplementary Figure 2. *gbb* overexpression in the fat body induces obesity and diabetic phenotypes.** (A) HFD increased *gbb* expression in the adult fat body of *Drosophila*. (B) *gbb* overexpression in the fat body (*DCG>gbb*), but not in the gut (*NP3084>gbb*) or muscle (*MHC>gbb*), increased the level of triglyceride. (C) *gbb* overexpression in the fat body (*DCG>gbb*) reduced the level of pAKT. (D) Overexpression of *gbb* mRNA in the fat body under the control of the fat body gene switch driver *pS106*$^{GS}$. (E) *Ex vivo* culture of *Drosophila* adult fat body with insulin stimulation. Data are presented as means ± s.e.m. from at least three independent experiments. *$P$<0.05.

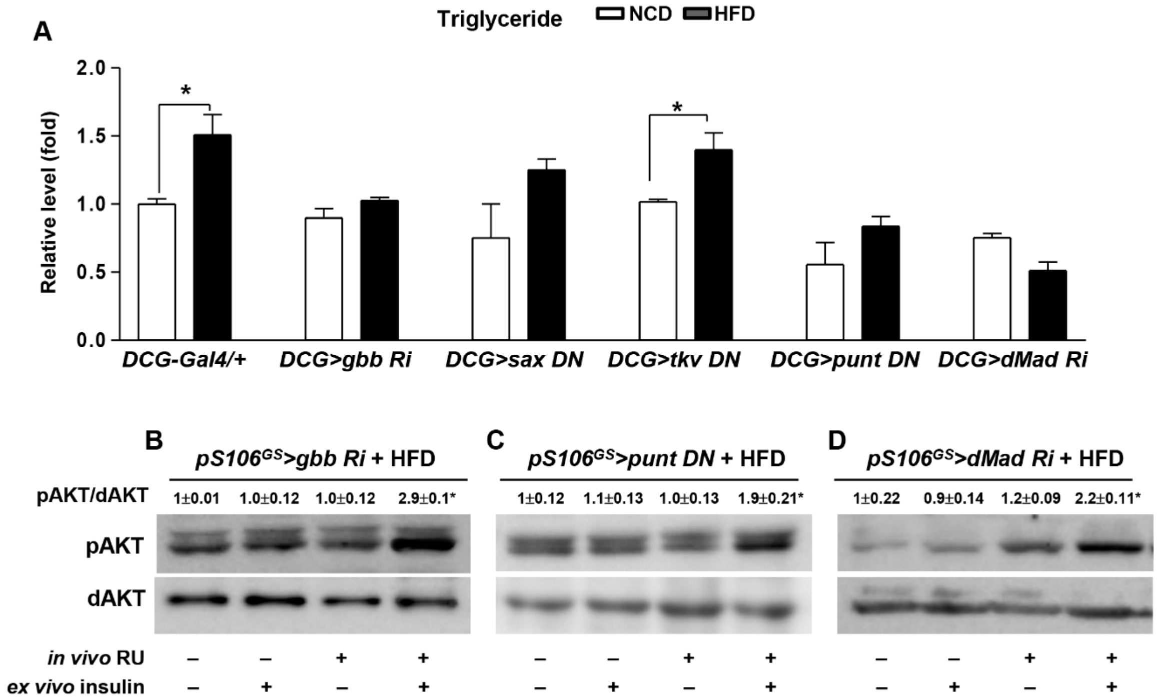


**Supplementary Figure 3. Inhibition of Gbb signaling rescues the reduced insulin signaling caused by HFD.** (A) The elevated triglyceride level in the HFD *DCG-Gal4/+* control was not observed in *DCG>gbb Ri*, *DCG>sax DN*, *DCG>punt DN*, and *DCG>dMad Ri*. (B–D) In the HFD condition, *ex vivo* insulin treatment did not activate pAKT in the fat body unless Gbb signaling was inhibited by *pS106*$^{GS}$*>gbb RNAi* +RU, *pS106*$^{GS}$*>punt DN* +RU, or *pS106*$^{GS}$*>dMad RNAi* +RU. Data are presented as means ± s.e.m. from at least three independent experiments. **P*<0.05.

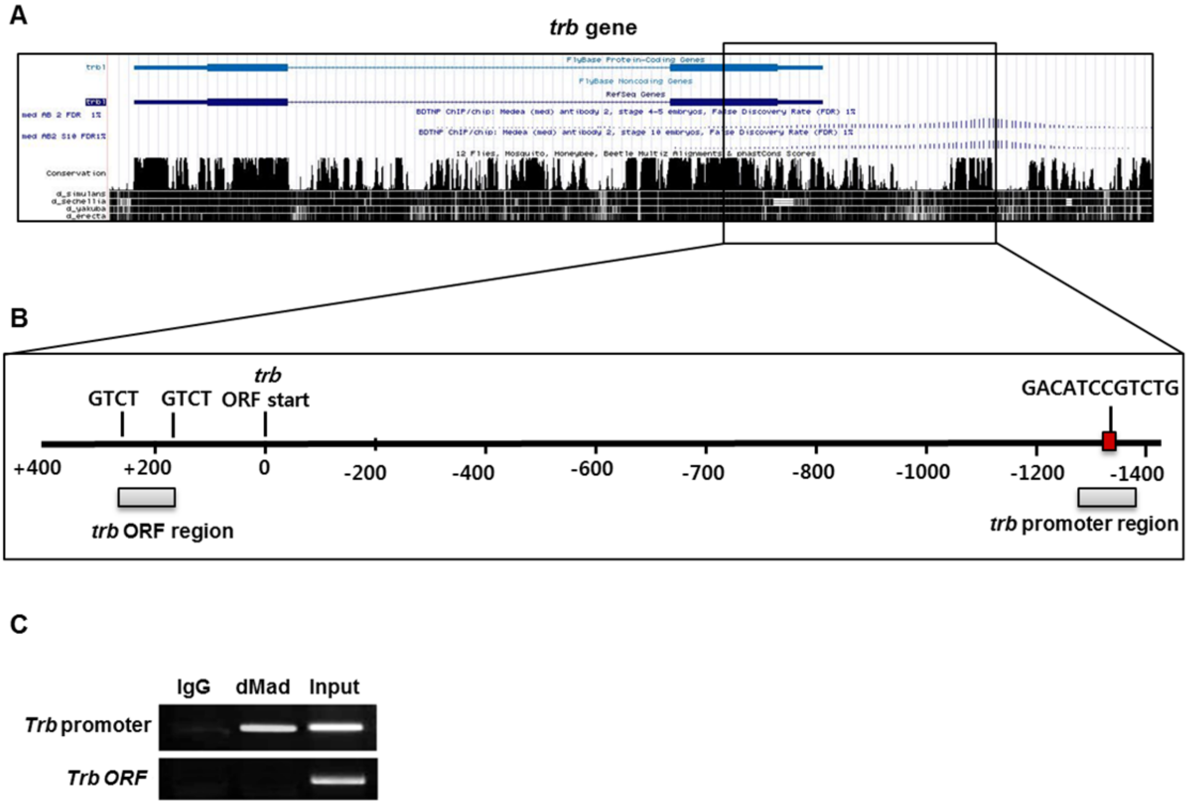


**Supplementary Figure 4. Promoter analysis of the *trb* gene.** (A) Genomic region of the *trb* gene. Boxes indicate the predicted promoter and ORF regions, which were used in the ChIP-PCR assay. (B) The *trb* promoter region, upstream of the transcriptional start site of the *trb* gene, contains repeats of the Mad-binding consensus sequence (GNCN or GTCT) and also contains Mad/Medea-binding sites identified in ChIP analyses performed by the Berkeley *Drosophila* Transcription Network Project (data were adapted and modified from the UCSC Genome Browser, http://genome.ucsc.edu). The *trb* ORF (coding region) also contains two Mad-binding sites (GTCT). (C) ChIP-PCR revealed that dMad associates with the putative *trb* promoter region but not the *trb* ORF.

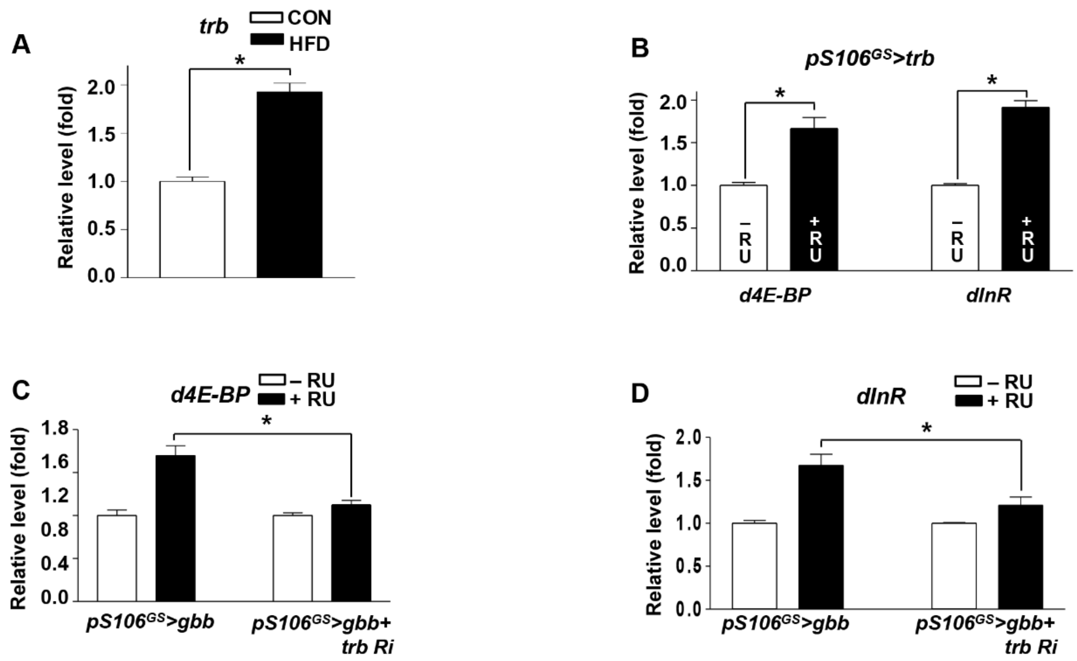


**Supplementary Figure 5. Inhibition of *trb* suppresses *gbb*-induced obesity and diabetic phenotype.** (A) Expression of *trb* was elevated in flies fed a HFD relative to those fed a normal control diet. (B) *trb*-overexpressing fat bodies (*pS106*$^{GS}$>*trb* +RU) increased the expression levels of *d4E-BP* and *dInR.* (C, D) Knockdown of *trb* in *gbb*-overexpressing fat bodies (*pS106*$^{GS}$>*gbb*+*trb RNAi* +RU) suppressed the elevated expression levels of *d4E-BP* and *dInR* observed in *pS106*$^{GS}$>*gbb* +RU. Data are presented as means ± s.e.m. from at least three independent experiments. **P*<0.05.

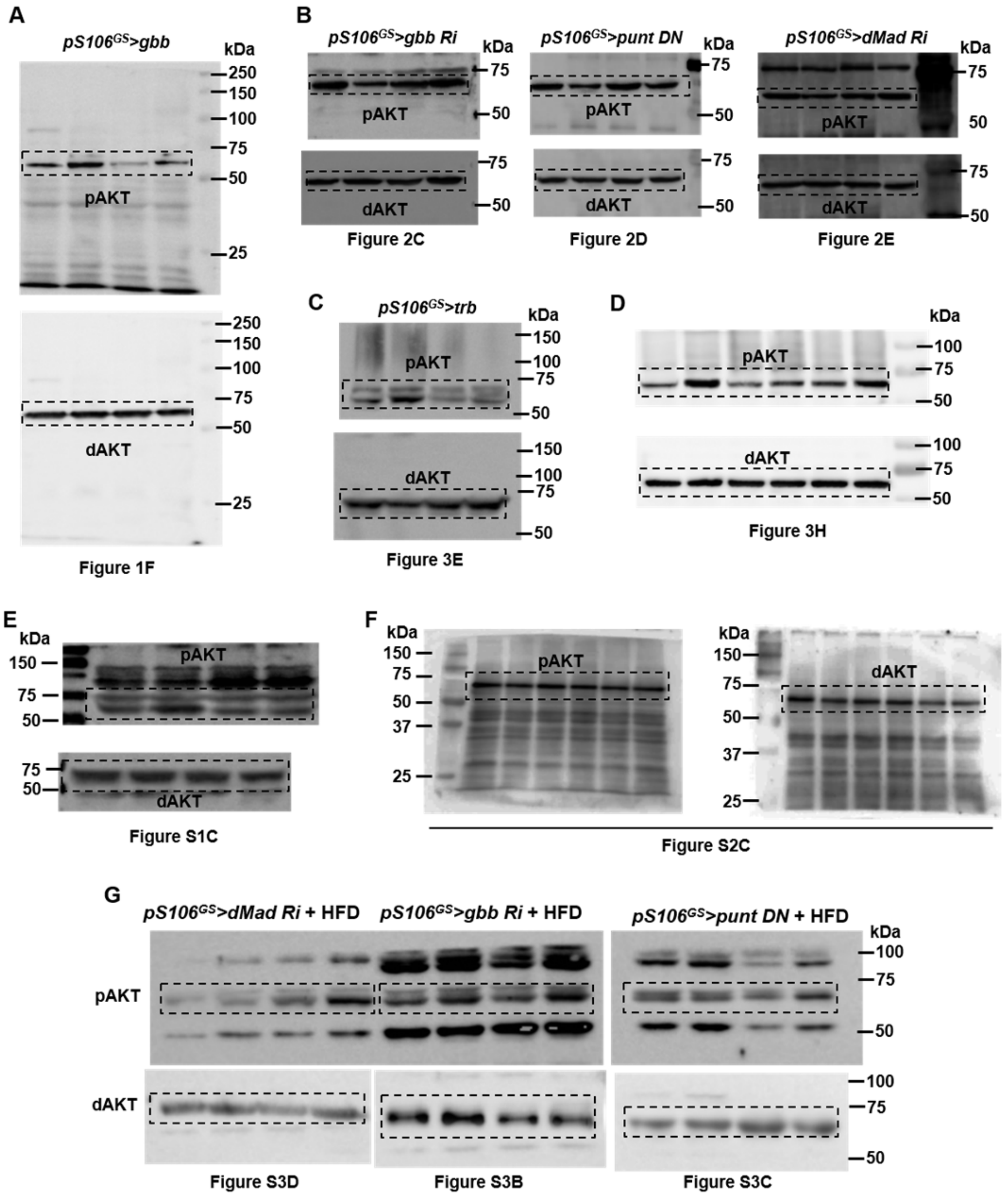


**Supplementary Figure 6. Complete scans of western blots shown in the figures.** (A-G) Dotted boxes indicate the parts that are shown in the figures. Please note that some membranes were cut into multiple strips prior to antibody detection to enable visualization of multiple antigens.

**Supplementary Table 1.** PCR Primers used in the qPCR analyses

| List | forward sequences | Reverse sequences |
|---|---|---|
| gbb, | CGTCGCCTGTGGTTCGACGT | CGGTCAGCCACTTGCCCTCG |
| dpp | TCGCGGTTTCTGCGGCCATAA | CTGGCTGACGAGCGATCGCG |
| dActivin, | ACAGCCTTGGCTCCCGGAAGT | CGCTACCGCAGTAACCCACCG |
| daw | TTTCATAGGGGCAGCGTACC | TCCATGGACTTCCTACAAACCA |
| myo | CGTGTGCAAACGAGACTTCC | CTGCGCTGCATTGCTACTTG |
| mav | GGCATATTCCGCCAAACGTC | CGGAGTTGATTTGGCTCGGA |
| d4E-BP | GATCACCAGGAAGGTTGTC | GGTCAATATGACCGAGAGAA |
| dInR | ACCTATTTAACCACAAGCGA | CTCGATAGTTCCAAGATTGC |
| PTP61F | CTCCTCTGAAATTAACCAGC | CGATGCCATTGCGGCCCCTT |
| dPten | AAAACGAAGCCACCAGAACTGGATT | ACAGATGTGCAGAGCGCAGCTG |
| trb | ACGCTGTCTAAGTCGGTGCG | AATGGCTCCACGCCAATGTC |
| RP49 | AGATCGTGAAGAAGCGCACCAAG | CACCAGGAACTTCTTGAATCCGG |

**Supplementary Table 2.** PCR Primers used in the ChIP and luciferase assays

| List | forward sequences | Reverse sequences |
|---|---|---|
| trb promoter | TCAGCTGCACTATTCCACCC | ATTGRGACCGCGAATGTGCT |
| trb ORF | AGTTCTTGCTCTCGCTACGTT | CGTTTCGCGAGTTCAGTGAG |
| pGL3-trb1.1 | AAGCTTTCACTGAACTCGCGAA ACGA | CTCGAGTCGTTTCGGACAGCTGCAAA |
| pGL3-trb1.4 | AAGCTTTCACTGAACTCGCGAA ACGA | CTCGAGTATTGTGACCGCGAATGTGC |
| pGL3-trb1.4ΔMbs_ external | TGCTAGCCCGGGCTCGAGTAT TGTGACCGC | TACCGGAATGCCAAGCTTTCACTGAA CTCG |
| pGL3-trb1.4ΔMbs_ internal | GCTCCTGTGTATCCTTTTAGTG GGAAATC | CAGTTGCAGCTGGCGCAAGGTGATT TCCCACTAAAAGGATACACA |
